# An ADAM10 Exosite Inhibitor Is Efficacious in an In Vivo Collagen-Induced Arthritis Model

**DOI:** 10.3390/ph17010087

**Published:** 2024-01-09

**Authors:** Juan Diez, Michael E. Selsted, Thomas D. Bannister, Dmitriy Minond

**Affiliations:** 1 Department of Pharmaceutical Sciences, Barry and Judy Silverman College of Pharmacy, Nova Southeastern University, 3321 College Avenue, Fort Lauderdale, FL 33314, USA; mdiezi@yahoo.com; 2 Department of Pathology and Laboratory Medicine, Keck School of Medicine, University of Southern California, 2011 Zonal Avenue, Los Angeles, CA 90089, USA; selsted@med.usc.edu; 3 Department of Molecular Medicine, The Herbert Wertheim UF Scripps Institute for Biomedical Innovation & Technology, 120 Scripps Way, Jupiter, FL 33458, USA; t.bannister@ufl.edu; 4 Rumbaugh-Goodwin Institute for Cancer Research, Nova Southeastern University, 3301 College Avenue, CCR r.605, Fort Lauderdale, FL 33314, USA

**Keywords:** rheumatoid arthritis, ADAM10, exosite inhibitor, in vivo efficacy, collagen-induced arthritis

## Abstract

Rheumatoid arthritis is a systemic autoimmune inflammatory disease that affects millions of people worldwide. There are multiple disease-modifying anti-rheumatic drugs available; however, many patients do not respond to any treatment. A disintegrin and metalloproteinase 10 has been suggested as a potential new target for RA due to its role in the release of multiple pro- and anti-inflammatory factors from cell surfaces. In the present study, we determined the pharmacokinetic parameters and in vivo efficacy of a compound CID3117694 from a novel class of non-zinc-binding inhibitors. Oral bioavailability was demonstrated in the blood and synovial fluid after a 10 mg/kg dose. To test efficacy, we established the collagen-induced arthritis model in mice. CID3117694 was administered orally at 10, 30, and 50 mg/kg/day for 28 days. CID3117694 was able to dose-dependently improve the disease score, decrease RA markers in the blood, and decrease signs of inflammation, hyperplasia, pannus formation, and cartilage erosion in the affected joints compared to the untreated control. Additionally, mice treated with CID 3117694 did not exhibit any clinical signs of distress, suggesting low toxicity. The results of this study suggest that the inhibition of ADAM10 exosite can be a viable therapeutic approach to RA.

## 1. Introduction

More than 1.3 million adults in the USA have been diagnosed with rheumatoid arthritis (RA) as of 2021 [1]. RA is a systemic autoimmune inflammatory disease that manifests in multiple joints of the body and leads to their degradation. The inflammatory process primarily affects the lining of the joints (the synovial membrane), leading to irreversible bone erosion, joint deformity, and loss of function, but can also affect other organs [2]. This inflammatory process is driven by an imbalance of inflammatory (e.g., TNFα, IL-6, IL-1) [3,4] and anti-inflammatory cytokines (e.g., IL-10, IL-Ra, soluble TNFR2, IL-11) [5,6,7] released from synoviocytes, which are cells that form the lining of the joints, and inflammatory cells (e.g., monocytes, neutrophils). The enzyme “a disintegrin and metalloproteinase 10” (ADAM10) has been identified as being involved in regulating the release (shedding) of cytokines from synoviocytes and other cell types [8,9].

The expression of the ADAM10 protein and mRNA was found to be elevated in the endothelial cells from RA synovial tissue, where it was shown to promote angiogenesis [9]. ADAM10 was also shown to be upregulated in the serum of RA patients, with levels correlating to the disease activity score (DAS28) [8]. The ADAM10 shedding of CD23 from B cells prompts surface-activated macrophages to release pro-inflammatory signals, which is proposed to lead to the progression of RA [10,11]. ADAM10 was found to positively mediate monocyte migration and adhesion to fibroblast-like synoviocytes (FLSs) [8], which were demonstrated to be a key cellular factor in RA [12]. The loss of FLSs’ ability to secrete ADAM10 results in the decreased production of CX3CL1 and VEGF [8]. The shedding of chemokines CX3CL1 and CXCL16 from human microvascular endothelial cells (HMVECs), FLS, and macrophages via ADAM10 leads to the recruitment of immune and inflammatory cells, thus promoting inflammation [9,13,14]. ADAM10 was also shown to release TNFα and mediate the release of IL-6 and IL-8 from FLS [13]. ADAM10 siRNA knockdown reduced RA-FLS proliferation, migration, and invasion, the secretion of VEGF-A and matrix metalloproteinase (MMP)-3 and -9 [13]. In vivo, ADAM10 siRNA reduced the RA score and VEGF-A, MMP-3, and MMP-9 levels in the collagen-induced arthritis model (CIA) in mice [13]. Based on these studies, ADAM10 represents an attractive target for RA drug development.

To the best of our knowledge, there are no selective ADAM10-targeted RA therapies on the market or in development. RTD-1, a macrocyclic peptide inhibitor of ADAM17, is currently being evaluated by Oryn Therapeutics [15,16,17]. Disease-modifying small molecule anti-rheumatic drugs (DMARDs) approved or under evaluation for therapy in RA include Janus kinase (JAK) inhibitors, TNF inhibitors, T cell co-stimulation blockers, IL-6 receptor inhibitors, promoters of B cell depletion, and interleukin 1 inhibitors. The ACR70 score for these treatments has shown enhanced efficacy over methotrexate monotherapy (70% improvement for responders), though the response rates are below 50% [18]. Therefore, there is still an unmet need for drugs that could improve upon ACR70 efficacy and (especially) its response rate either alone or in combination with approved drugs. Based on this need, small molecule inhibitors of ADAM10 can be a novel and effective prevention or treatment therapy option. 

Our group has previously reported the discovery of the novel selective exosite-binding inhibitor of ADAM10, CID 3117694 [19]. In the studies presented herein, we report the results of the efficacy testing of CID 3117694 in the mouse CIA model of RA.

## 2. Results

Database search. CID 3117694 profiling suggests a high level of ADAM10 target selectivity. Searches of the PubChem database for the biological activity of CID 3117694 revealed it to be inactive in 524 reported bioassays and active against only 3 targets, with ADAM10 being the top target (PubChem AID 743338). A second target was the cardiac ion channel hERG, though it is not an hERG inhibitor (a risk for cardiotoxicity). Rather, CID 3117694 was shown to be cardioprotective, diminishing the effects of pro-arrhythmic agents (PubChem AID 1511, no EC50 value reported). The third known target of this molecule is DNA polymerase β (PubChem AID 485314), where CID3117694 is only very weakly active (IC50 = 79 µM). Among a variety of drug safety targets studied, it is inactive against adrenergic (ADRB2), muscarinic (CHRM1), and opioid receptors (OPRK1, OPRM1, and OPRD1) (21). It lacks T-cell-based effects, as reflected by its inactivity in an assay for activators of T-cell receptors (see PubChem bioassay 504894). It also does not affect caspase 8 activity. These data show that CID 3117694 is not target-promiscuous, which is a feature that should translate into low off-target in vivo toxicity and is an attribute that is particularly noteworthy for potential RA therapeutic leads and, in general, is quite rare for inhibitors of Zinc metalloproteases.

**Figure 1:**
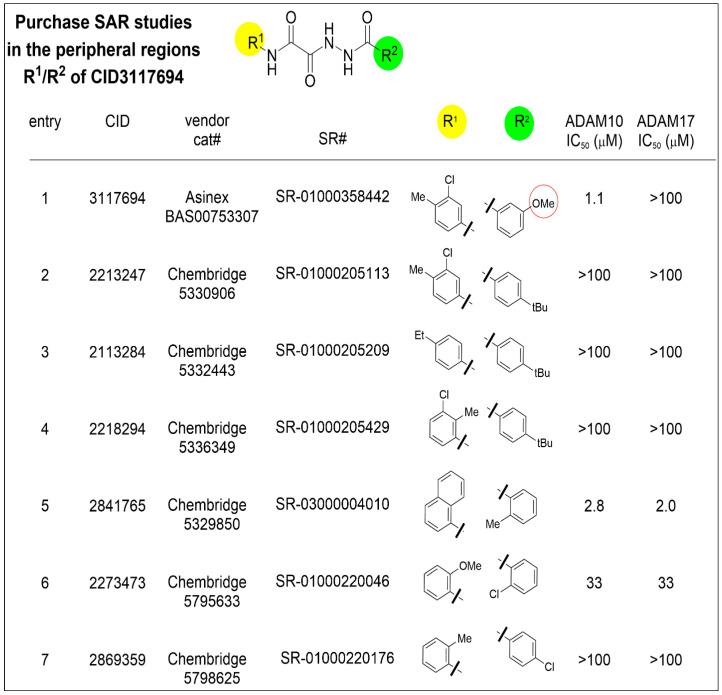
Structural analogs of CID 3117694 were acquired from Chembridge, with activity measured as indicated. Yellow circle—substitutions tested in R1 position, green circles—substitutions tested in R2 position, red circle—methoxy group in R2 position of CID 3117694.

CID 3117694 structure–activity relationship (SAR) study. We purchased several commercially available analogs of CID 3117694 to determine the features important for ADAM10 binding. Substitutions of the aryl meta methoxy moiety in the R2 position (circled, Figure 1, entry 1) for para tert-butyl, combined with various substitutions in the R1 position (entries 2–4), were not tolerated and resulted in a total loss of activity against ADAM10, suggesting the importance of this moiety. The introduction of chlorine in the para position of R2 (Figure 1, entry 7) did not rescue the activity against ADAM10, suggesting that future SAR studies should preserve the methoxy or a similar group in the meta position. The simultaneous introduction of an ortho methyl in R2 and the 2-naphthyl group as R1 retrieved the activity of ADAM10; however, selectivity against ADAM17 was lost (Figure 1, entry 5). Similar to entry 5, entry 6 was substituted for o-Cl for m-OMe in R2 and an o-OMe in the R1 phenyl and showed no selectivity and reduced affinity. These SAR data can guide our future efforts for the optimization of CID 3117694.

**Figure 2:**
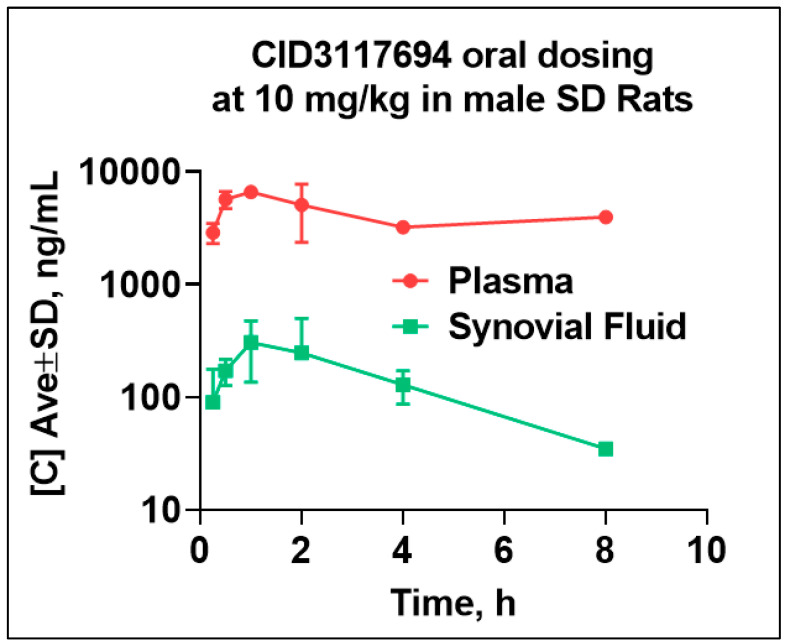
CID 3117694 penetrates the synovium at a therapeutically relevant concentration. CID 3117694 was formulated in 10% DMSO, 40% PEG400, 30% PG, 20% H_2_O at 2 mg/mL and administered via an intragastric (ig) gavage to six Sprague Dawley male rats at 10 mg/kg. The 24 h time point is not shown due to the compound being below the limit of quantitation (LOQ).

Pharmacokinetics of CID 3117694 in Sprague Dawley male rats. The CID 3117694 structure was confirmed using H1 NMR (Figure S1), and it was determined to be >95% pure via HPLC (Figure S2). To determine if CID 3117694 can be used in in vivo RA efficacy model studies, we conducted an in vivo pharmacokinetic (PK) study. CID 3117694 was formulated in 10% DMSO, 40% PEG400, 30% PG, and 20% H_2_O at 2 mg/mL and administered via oral gavage to Sprague Dawley male rats at 10 mg/kg. Blood and synovial fluid were collected at 15 min, 30 min, 1, 2, 4, 8, and 24 h and quantitated using LC/MS. PK parameters were calculated using WinNonLin 8.3.4 software. As can be seen in Figure 2 and Table 1, Cmax (maximal concentration) in plasma and synovial fluid (SF) was reached 1 hr after administration. Cmax in SF and plasma was 849 nM and 18.4 µM, respectively. The Ki value of CID 3117694 for ADAM10 inhibition was 883 nM (19), suggesting that this compound penetrates the synovium to reach a potentially therapeutically relevant concentration. As mentioned above, CID 3117694 was tested against multiple cell lines at a high concentration (10 µM and up to 100 µM in some cases) and showed no effect on cellular viability (PubChem Assay IDs 1794763-1794787) [19,20]. This observation, in combination with the above-mentioned evidence for minimal off-target binding, suggests that a suitable safety margin may exist for in vivo use.

In vivo efficacy in the CIA mouse model. Encouraged by the results of the PK study, we proceeded to an efficacy and dose-finding study using the collagen-induced arthritis (CIA) model. To enable the delivery of CID 3117694 at doses 10 mg/kg, 30 mg/kg, and even 50 mg/kg doses, we identified a modified formulation that used hydroxy–propyl–methyl cellulose (HPMC) to augment solubility.

**Figure 3:**
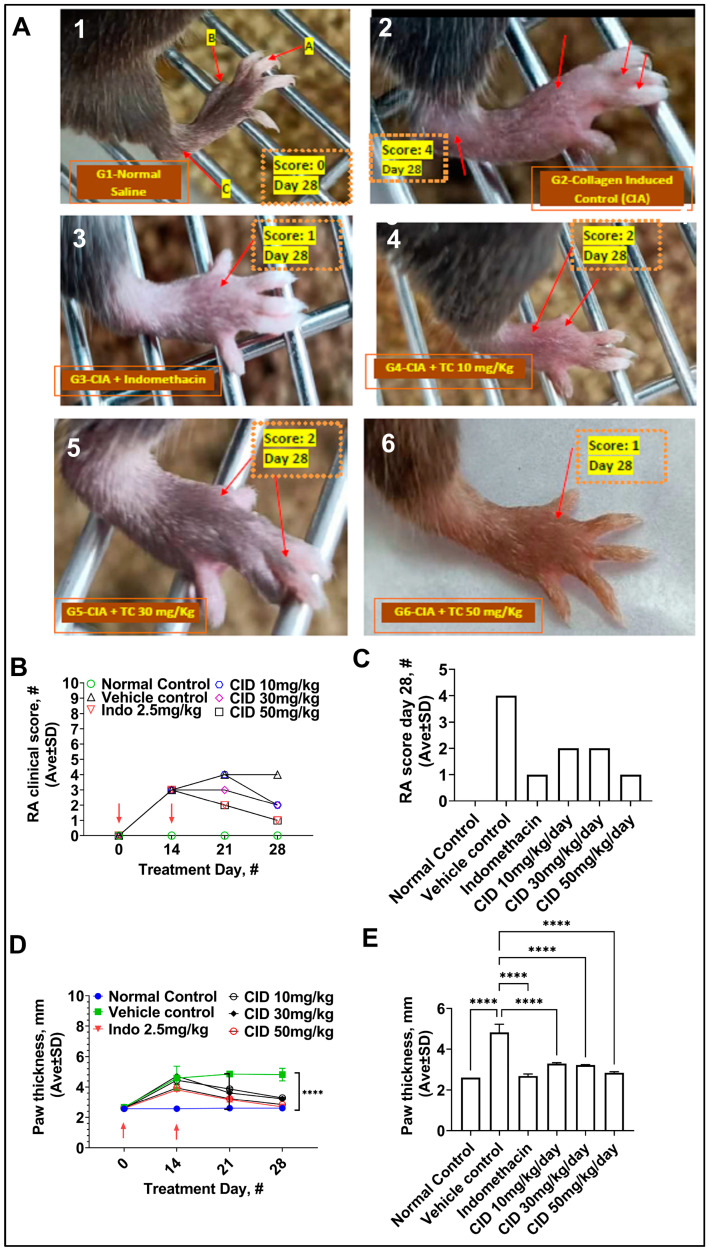
CID 3117694 lowers the RA clinical score. (**A**) Clinical score assignment on the last day of treatment (day 28). Panel 1—normal control. Red arrows point to the joints used for clinical score assignment (A—interphalangeal, B—metacarpophalangeal, C—carpal and tarsal), panel 2—disease vehicle control, panel 3—disease treated with 2.5 mg/kg indomethacin, panel 4—disease treated with 10 mg/kg CID 3117694, panel 5—disease treated with 30 mg/kg CID 3117694, panel 6—disease treated with 50 mg/kg CID 3117694. (**B**) RA clinical scores over time and (**C**) on day 28. Red arrows indicate the day of RA induction and booster injection. Note—all clinical scores were uniform within each group, resulting in SD = 0, which did not allow us to perform a statistical significance analysis. (**D**) Paw thickness measurements over the course of the treatment demonstrated time-dependent improvement in all mice treated with CID 3117694; (**E**) Paw thickness measurements on the last day of treatment (day 28) showed no significant differences between the vehicle control and treatment groups and dose-dependent improvement was seen in all mice treated with CID 3117694. ****—*p*-value < 0.001. Data shown are the mean ± SD (*n* = 6) analyzed using a one-way ANOVA followed by Dunnett’s test compared with a vehicle control. Three male and three female 6–8-week-old C57 BL/6 mice from Charles River were used for this experiment.

We began administering both the control and test compound simultaneously with RA induction on day 0. Three male and three female 6–8-week-old C57 BL/6 mice from Charles River were used for this experiment. On day 14 after RA induction, the clinical score was three for all groups, with the exception of normal control, suggesting that no test dose was effective at this time point in preventing disease progression (Figure 3A). By day 21, however, a divergence in efficacy was first noted. The disease score for the vehicle control and for a 10 mg/kg dose of CID 3117694 increased to 4, while indomethacin and a 50 mg/kg dose of CID 3117694 lowered the score to 2. The 30 mg/kg CID 3117694 dose animals were scored at three. The 28-day timepoint showed more pronounced effects as follows: the 10 mg/kg and 30 mg/kg doses of CID 3117694 lowered the disease score to 2, while indomethacin and a 50 mg/kg dose of CID 3117694 lowered the disease score to 1 (Figure 3A–C).

**Figure 4:**
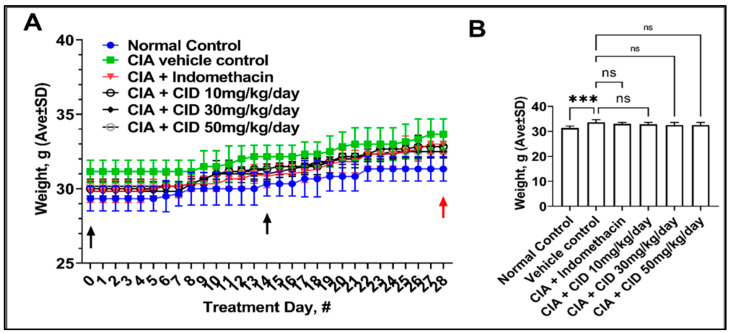
C57BL/6 mice do not exhibit weight loss after treatment with CID 3117694. (**A**) Body weight measurements over the course of the treatment and (**B**) the last day demonstrated overall weight gain in all mice. Mice were treated perorally with indomethacin and CID at 3117694 3×/week for 28 days. Black arrows indicate the days of RA induction and booster injections. The red arrow indicates the day the mice were euthanized. ***—*p*-value < 0.001, ns—no significance (*p*-value > 0.05). Data shown are the mean ± SD (*n* = 6) analyzed using a one-way ANOVA followed by Dunnett’s test compared with a vehicle control. Three male and three female 6–8-week-old C57 BL/6 mice from Charles River were used for this experiment.

Targeting zinc-binding metalloproteases has historically been a difficult drug development strategy, especially when using Zn-binding small molecules, primarily due to the challenge of overcoming the toxicity arising from off-target binding (22). We felt that CID 3117694, which does not bind with zinc, may have advantages with respect to target fidelity and a lowered demonstrated toxicity in animals. Body weight, a gross indicator of animal distress and toxicity, was monitored during the in vivo study, along with any other behavioral signs of distress. As evidenced by Figure 4A,B, no significant differences in body weight between the vehicle control and treatment groups were observed, suggesting that CID 3117694 did not impact the feeding behavior, which could arise from many effects, including the induction of nausea. We also observed no clinical signs of distress (e.g., change in behavior or appearance) that can typically be attributed to drug toxicity.

A hallmark of RA, paw swelling, was monitored on days 1, 14, 21, and 28 of the study (Figure 3C,D). CID 3117694 was able to time- and dose-dependently decrease paw swelling. Moreover, the highest dose of CID 3117694, 50 mg/kg/day, decreased the swelling to the level of the normal control (Figure 3D).

**Figure 5:**
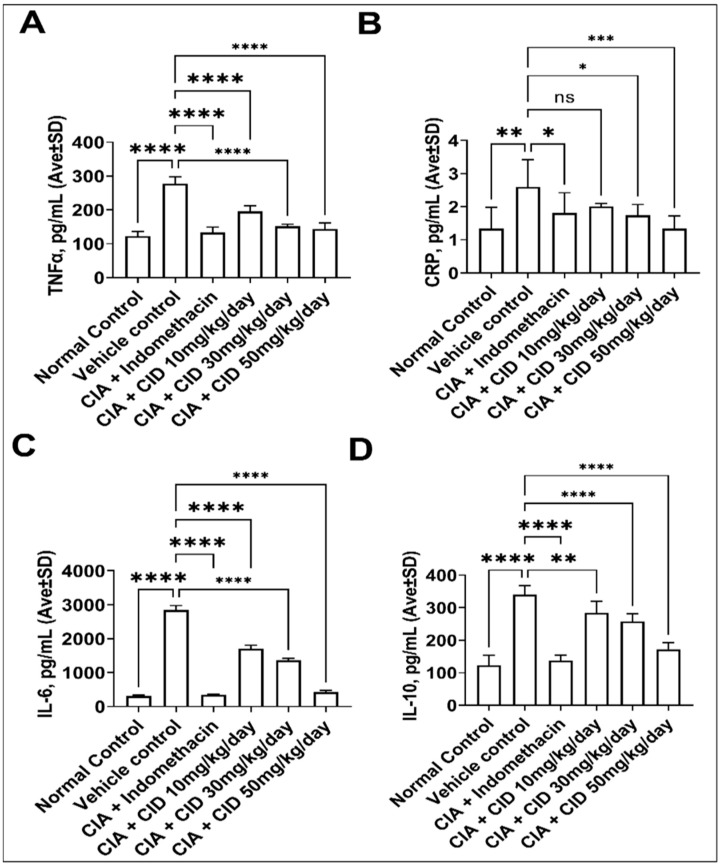
CID 3117694 is efficacious at decreasing the molecular markers of RA in the collagen-induced model. (**A**) CID 3117694 lowers the concentration of TNFα in the blood of arthritic mice at all tested doses; (**B**) CID 3117694 lowers the concentration of CRP in the blood of arthritic mice at 30 and 50 mg/kg; (**C**) CID 3117694 lowers the concentration of IL-6 in the blood of arthritic mice at all tested doses; (**D**) CID 3117694 lowers concentration of IL-10 in the blood of arthritic mice at all tested doses. ns—no significance, *—*p*-value < 0.05, **—*p*-value < 0.01, ***—*p*-value < 0.005, ****—*p*-value < 0.001. Data show the mean ± SD (*n* = 6) analyzed using a one-way ANOVA followed by Dunnett’s test compared with a vehicle control. Three male and three female 6–8-week-old C57 BL/6 mice from Charles River were used for this experiment.

To determine the effect of CID 3117694 on molecular markers of inflammation of RA in the blood, we performed ELISA assays for TNFα, the C-reactive protein (CRP), IL-6, and IL-10. As can be seen in Figure 5, CID 3117694 dose-dependently decreased all four markers. Noteworthy, the highest tested dose (50 mg/kg/day) decreased the markers to the level of the healthy control.

**Figure 6:**
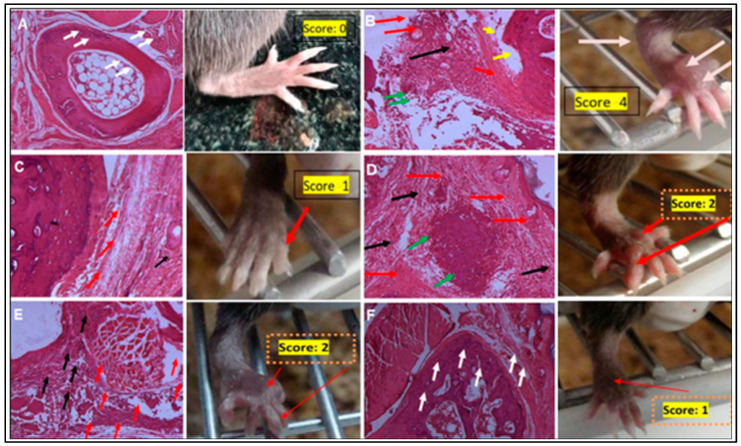
CID 3117694 decreases histopathological markers of RA. (**A**) Normal control; (**B**) Vehicle control; (**C**) Indomethacin 2.5 mg/kg/day; (**D**) 10 mg/kg/day CID 3117694; (**E**) 30 mg/kg/day CID 3117694; (**F**) 50 mg/kg/day CID 3117694. The images shown are representative of H & E-stained sagittal sections of the arthritis joint of the hind paw examined via light microscopy at 100× magnification. Six slides per test group were examined. White arrows: normal healthy bone and cartilage of arthritis joint; red arrows: synovial inflammation; black arrows: hyperplasia; green arrows: pannus formation; yellow arrows—cartilage erosion. Three male and three female 6–8-week-old C57 BL/6 mice from Charles River were used for this experiment.

We also evaluated the effect of CID 3117694 on the histology of articular joints affected by RA. Histopathological examination revealed significant inflammation, hyperplasia, pannus formation, and cartilage and bone erosion of the untreated RA mice compared to healthy mice consistent with RA scores (Figure 6A,B). Indomethacin-treated mice exhibited only residual inflammation and hyperplasia, which was significantly reduced compared to untreated RA mice (Figure 6C). 10 mg/kg/day of CID 3117694 did not have a pronounced effect on RA markers (Figure 6D). A total of 30 mg/kg/day of CID 3117694 reduced erosion but not the rest of the symptoms (Figure 6E), while 50 mg/kg/day of CID 3117694 showed a significant reduction in RA hallmarks in most samples (Figure 6F) comparable to indomethacin. Overall, CID 3117694 dose-dependently alleviated histopathological hallmarks of RA, suggesting its potential for therapy.

## 3. Discussion

As mentioned in the Results, a 10 mg/kg oral dose of CID 3117694 exhibited Cmax in the SF and plasma of 849 nM and 18.4 µM, respectively. Given that the K_i_ value of CID 3117694 for ADAM10 inhibition is 883 nM (19), we expected to see efficacy in the in vivo model at the same dose. Indeed, the 10 mg/kg dose improved the RA score, paw thickness, TNFα, IL-6, and IL-10 RA markers, suggesting target modulation. It is important to note that CID 3117694 is orally bioavailable and was present in the blood at a steady-state concentration, which is necessary for effective treatment for at least 8 h. This suggests the good metabolic stability and clearance rate of CID 3117694. By contrast, the CID 3117694 concentration in SF decreased below Ki levels 2 h post-treatment. The reason for this difference and the implication of therapy is not clear and requires additional study. One possibility involves a higher rate at which CID 3117694 is taken up by the cells of the synovium. This also provides a foundation for future in vivo studies using CID 3117694 in RA and other diseases.

The highest dose of CID 3117694, 50 mg/kg, had an effect on all the tested parameters, with the exception of histopathology, similar to 2.5 mg/kg of Indomethacin. Both 50 mg/kg CID 3117694 and 2.5 mg/kg of Indomethacin improved the RA score and paw thickness almost to the level of healthy controls (score 1 and 0, respectively), whereas corresponding blood marker levels were the same as that of the healthy controls. The histopathology of mice treated with 2.5 mg/kg of indomethacin revealed the presence of inflammation and hyperplasia, whereas 50 mg/kg CID 3117694 exhibited healthy bone and cartilage similar to the healthy controls. The differences can be attributed to the different mechanisms of action. Hyperplasia is the overgrowth of synovial and immune cells in joints leading to pannus formation, which is a thick, swollen synovial membrane with granulating tissue consisting of myofibroblast, fibroblast, and inflammatory cells [21]. Hyperplasia formation is macrophage-dependent [21], whereas indomethacin is an NSAID that works by inhibiting enzymes COX-1 and COX-2 expressed on platelets. ADAM10 is expressed on macrophages, whereas COX-1 and COX-2 are not [22]; therefore, it is possible that hyperplasia and inflammation that is not inhibited by indomethacin are mediated by macrophages infiltrating the synovial joints. We demonstrated previously that CID 3117694 can inhibit the recruitment of PBMCs and neutrophils [19], suggesting that it can also inhibit the recruitment of macrophages to the synovial joints.

As discussed in the Introduction, ADAM10 has been shown to shed multiple substrates from the surface of inflammatory cells implicated in RA development and progression. Some of these substrates (e.g., TNFα, IL6) can also be shed by its closest analog, ADAM17 (also known as the TNFα converting enzyme (TACE) [23,24], and, possibly, by other enzymes. The data presented here indicate that the inhibition of ADAM10 is sufficient to stop the RA-induced shedding of TNFα. 

ADAM10 both sheds and mediates the shedding of multiple proteins from the cell surface [25,26,27,28,29,30], some of which have a known role in RA, such as TNFα and IL6 [13], CXCL16 [31], to name a few. The present study demonstrates the ability of the ADAM10-selective inhibitor CID 3117694 to decrease levels of some of these molecules in the blood, suggesting the abrogation of shedding and, therefore, successful target modulation. One potential implication of this result is an ability to use these molecules as ADAM10 pharmacodynamic (PD) markers in future RA pre-clinical and clinical studies.

CID 3117694 is an exosite-targeting inhibitor that exhibits unusual substrate selectivity in in vitro experiments [19], suggesting that not all ADAM10 substrates are protected by the administration of this compound in vivo. In the present proof-of-principle study, we only assessed levels of some of the RA-relevant inflammatory molecules. To ascertain the full profile of substrates protected by CID 3117694, a larger proteomic study is needed. However, despite a potentially limited substrate-protective effect, CID 3117694 exhibited disease-modifying properties that warrant further studies.

A potential limitation of this study is the lack of detailed knowledge about how all ADAM10 substrates affect RA progression. As discussed in the Introduction, ADAM10 sheds both pro- and anti-inflammatory molecules, and a compound-specific shedding inhibition profile could help determine the relative importance of these substrates in RA progression and identify opportunities for safe therapeutic intervention. While no signs of toxicity attributable to CID 3117694 were observed during the 28-day CIA study, more extensive toxicological evaluations are required to gauge the effects of longer periods of ADAM10 inhibitor administration.

## 4. Materials and Methods

Reagents and Materials. CID3117694 was obtained from AKos Consulting & Solutions (Lörrach, Germany). All common chemicals and buffers were obtained from VWR. ADAM10 and ADAM17 enzymes were from R & D Systems (Minneapolis, MN, USA). The synthesis of ADAM10 and ADAM17 substrates was described previously by us [19]. 

ADAM10 and ADAM17 assays. Both assays were performed as published previously by us [19]. Briefly, both assays followed the same general protocol. In total, 5 μL of the 2× enzyme solution (20 nM) in an assay buffer (10 mM Hepes, 0.001% Brij-35, pH 7.5) were added to solid bottom black 384 plates (Nunc, ThermoFisher, Waltham, MA, USA, cat# 264705). Next, test compounds and pharmacological controls were added to the corresponding wells using a 384-pin tool device (V & P Scientific, San Diego, CA, USA). After 60 min of incubation at RT, the reactions were started via the addition of 5 μL of the 2× solutions of glycosylated substrate DM2 (20 μM). The reactions were incubated at RT for 2 h, after which the fluorescence was measured using a Biotek H1 multimode microplate imager (λ_excitation_ = 360 nm, λ_emission_ = 460 nm). IC_50_ values were calculated by fitting normalized data to the sigmoidal log vs. the response equation utilizing a non-linear regression analysis from GraphPad Prizm 9.

Pharmacokinetics of CID 3117694. All animal studies were conducted in accordance with NSU IACUC guidelines. Study protocol #2021.04.DM2 was approved using NSU IACUC before the commencement of the studies. CID 3117694 was formulated in 10% DMSO, 40% PEG400, 30% PG, and 20% H_2_O at 2 mg/mL and administered via an intragastric (ig) gavage to six Sprague Dawley male rats at 10 mg/kg. The animals were anesthetized using isoflurane. Blood and synovial fluid were collected at 15 min, 30 min, 1 h, 2 h, 4 h, 8 h, and 24 h after the administration of CID 3117694. Synovial fluid (SF) was collected using a 23-gauge needle connected to a mini peristaltic pump via perfusion tubing. Sterile saline was infused at a constant rate of 100 μL/min^−1^. After the infusion of 100 μL of the vehicle (sterile saline), the outflow tubing was connected to the 25-gauge needle to minimize pressure build-up within the joint space. The fluid was infused and withdrawn at a constant rate until a 250 μL basal sample was collected in a 1.5 mL centrifuge tube. Samples were immediately frozen at −20 °C. Blood was collected using a 25 G needle directly from the heart. The samples were prepared for LC-MS/MS analysis using the acetonitrile protein precipitation technique. Briefly, blank plasma and subject samples (plasma and SF samples) were retrieved from the deep freezer and allowed to thaw. The calibration curve standards (10 to 10,000 μg/mL of CID 3117694) and quality control samples were prepared using blank plasma. The thawed samples were vortexed to ensure the complete mixing of the contents. A total of 50 µL of each sample was transferred to the vials, and 200 µL of an Internal Standard (IS, telmisartan) mixed with acetonitrile was added to all the samples and vortexed. Samples were kept on the shaker for 5 min to ensure complete mixing of contents. The samples were centrifuged at 4000 rpm and 20 °C for 10 min, and the supernatant was transferred into auto-injector vials and loaded into the auto-sampler. A total of 5 µL was injected onto the API-4500 Q TRAP LC-MS/MS system. The samples were separated on a Phenomenex, Synergi C18 4.6 mm × 50 mm, 4 µm HPLC column using a 10 mM ammonium acetate + 0.1% Formic acid buffer as solvent A and 100% Acetonitrile as solvent B. PK parameters were quantitated using WinNonLin (Certara, Princeton, NJ, USA).

In vivo efficacy in the CIA mouse model. All animal studies were conducted in accordance with NSU IACUC guidelines. Study protocol #2021.04.DM5 was approved by NSU IACUC before the commencement of the studies. CID 3117694 and indomethacin were ground with a small amount of the dispersing agent (0.5% HPMC (3 cP)–0.5% Tween 80) using a mortar and a pestle and mixed repeatedly to form a smooth paste. The paste was moved to a graduated cylinder and filled up to the required volume with the dispersing agent to form a suspension, as described elsewhere [32]. The test compound (10, 30, and 50 mg/kg) and indomethacin (2.5 mg/kg) were administered with a mouse gavage needle (gauge no: 18, BD, catalog number: 653902) through the oral route as a suspension with a 5 mL/kg dose volume (from day 0 to day 28).

In this study, 6–8-week-old C57 BL/6 mice from Charles River (male and female) were used. Animals were randomly assigned to one of the following six groups: (1) normal (non-RA control), (2) RA control untreated, (3) RA control treated with 2.5 mg/kg/day indomethacin, and 3 test groups (CID 3117694 (10 mg/kg/day, 30 mg/kg/day, and 50 mg/kg/day)) to provide an estimate for the lead compound’s efficacy range. Each group had three males and three females assigned to the control for gender-based differences.

Arthritis was induced in the right paws of the mice under anaesthesia. The left paw of the mice was used as a control. A total of 10 µL collagen from bovine (type II collagen) nasal septum emulsion (2 mg/mL) was injected into the right knee joint with a glass Hamilton syringe and 25 G needles for intraarticular (i.a.) injection, followed by a total of 100 µL Complete Freund’s adjuvant (CFA) emulsion was injected at the back of mice using a syringe with 25 G needles subcutaneously (s.c.). On day 14 after the first injection, the mice were injected, i.e., with 10 µL of collagen from the bovine nasal septum (2 mg/kg) and s.c. with a 100 µL CFA emulsion for a boost injection.

Arthritis progression was monitored using a clinical arthritis scoring system (0–4), and paw swelling was measured with the help of a plethysmograph on days 0, 14, 21, and 28 after the induction of arthritis. To assign a clinical score, interphalangeal, metacarpophalangeal, carpal, and tarsal joints were observed for swelling and redness. Normal joints were assigned a score of 0; in cases where only one joint type was affected, the score was 1; in cases where two joint types were affected, the score was 2; in cases where three joint types were affected, the score was 3; the maximal redness and swelling of the entire paw with the loss of an anatomic definition resulted in a score of 4.

After the administration of a primary dose of collagen from the bovine nasal septum, CFA scoring was assessed. Measurements were taken on days 0, 14, 21, and 28 after the induction of arthritis in induced paws (right paw) and non-arthritis paws (left paw). CID 3117694 (10 mg/kg/day, 30 mg/kg/day, and 50 mg/kg/day) and pharmacological control (indomethacin 2.5 mg/kg/day) treatment were given every day for 28 days beginning from day 1 via oral gavage in hydroxy-propyl-methyl cellulose (HPMC) suspension.

Mice were weighed and observed every day, and notes were made on the signs of distress. More specifically, mice were observed for posture, vocalization, ease of handling, lacrimation, chromodacryorrhea, salivation, coat condition, unsupported rearing, arousal, piloerection, motor movements, diarrhea, tail pinch reaction, constipation, and death.

The sample size for therapeutic evaluation was calculated using power analysis based on experimental data from similar studies. In a two-sided test and setting the α value at 0.05 and desired power at 0.95, the sample size turned out to be 5 per group. We also expected deaths during the experiments; therefore, we used the formula Nt = N/1-q, where Nt is the number of mice initially used, N is the number required at the end of the study, and q is the proportion of attrition, which is generally 10%. Thus, at the end of the study period, we expected to have data from at least 6 mice per group, which is enough to attain statistical significance if it exists. Data were analyzed using a one-way ANOVA followed by Dunnett’s test compared with the vehicle control. At the end of the study, the mice were anesthetized and sacrificed using CO_2_ euthanasia. The hind paws of the mice were subjected to a histopathological examination, and the serum was analyzed for CRP, TNF-α, IL-6, and IL-10, as described in the below sections.

All data were expressed as the mean ± SEM and statistically analyzed using IBM-SPSS version 20 software using one-way ANOVA followed by a post hoc Dunnett t-test at different variance levels. A *p*-value of 0.05 or less was considered statistically significant.

RA serum marker analysis. Blood was collected from the tail vein, and 100 µL was used for the assays. All assays were conducted using the manufacturer’s instructions for the respective assay kits. The following assay kits were purchased: Mouse TNF-α GENLISA™ ELISA (Krishgen BioSystems cat#KB2145, Mumbai, India), Mouse IL-10 GENLISA™ ELISA (Krishgen BioSystems cat#KB2072), Mouse hsC-Reactive Protein, hs-CRP GENLISA™ ELISA (Krishgen BioSystems cat#KLM0318), Mouse IL-6 GENLISA™ ELISA (Krishgen BioSystems cat#KB2068).

Histology. Limbs from euthanized animals were preserved in buffered formalin, decalcified, embedded, sectioned, and stained with hematoxylin and eosin (H & E). Microscopic images were acquired using the Lx 400 microscope (Labomed, Fremont, CA, USA).

## 5. Conclusions

CID 3117694 demonstrated efficacy in the treatment of CIA mice as evidenced by clinical, molecular, and histological markers of RA. Its excellent selectivity profile against related metzincins and limited off-target interactions suggests that its mechanism of action is based primarily on ADAM10 inhibition. This, in turn, suggests that ADAM10 exosite inhibition can potentially be a viable therapeutic approach for the treatment of RA. Additionally, CID 3117694 can be a useful tool to study the role of ADAM10 in various in vitro and in vivo models of diseases.

## Figures and Tables

**Table 1:** Results of the pharmacokinetic studies of CID 3117694 in male Sprague Dawley rats. SF—synovial fluid.

	**Plasma**	**SF**	**Concentration**		**CID 3117694 K_i_, M/L**
Dose, (mg/kg)	10		**Plasma, M/L**	**SF, M/L**	
Cmax, (ng/mL)	6643.3	307	1.84 × 10^−5^	8.49 × 10^−7^	8.83 × 10^−7^
Tmax (h)	1	1	N/A		
AUC0-last, (ng∗mL/h)	33,068.3	1100.2			
AUC0-inf, (ng∗mL/h)	173,161.3	1122.5			

## Data Availability

All data generated or analyzed during this study are included in this published article.

## Supplementary Materials

The following supporting information can be downloaded at: https://www.mdpi.com/article/10.3390/ph17010087/s1, Figure S1: Structural characterization of the batch of CID 3117694 synthesized for this study via H1 NMR confirms the identity of the compound; Figure S2: LC/MS/MS characterization of the batch of CID 3117694 synthesized for this study confirms its purity and correct molecular weight.
