# Supplementary material for: An ADAM10 Exosite Inhibitor Is Efficacious in an In Vivo Collagen-Induced Arthritis Model
Source: Pharmaceuticals (Basel). 2024 Jan 9;17(1):87. doi: 10.3390/ph17010087 (PMC10819767; doi:10.3390/ph17010087)
Supplement: Supplementary file 1 [file pharmaceuticals-17-00087-s001.zip › pharmaceuticals-2657811-supplementary.pdf]

Title: An ADAM10 exosite inhibitor is efficacious in an *in vivo* collagen-induced arthritis model

Authors: Juan Diez[1], Michael E. Selsted[2], Thomas D. Bannister[3], Dmitriy Minond[1,4*]

Affiliations:

[1] College of Pharmacy, Department of Pharmaceutical Sciences, Nova Southeastern University, 3321 College Avenue, Fort Lauderdale, FL, USA 33314

[2] Department of Pathology and Laboratory Medicine, Keck School of Medicine of the University of Southern California, 2011 Zonal Ave., Los Angeles, CA 90089

[3] Department of Molecular Medicine, The Herbert Wertheim UF Scripps Institute for Biomedical Innovation & Technology, 120 Scripps Way, Jupiter, FL, USA 33458

[4] Rumbaugh-Goodwin Institute for Cancer Research, Nova Southeastern University, 3301 College Avenue, CCR r.605, Fort Lauderdale, FL, USA 33314.

* Corresponding author. Email: dminond@nova.edu

One Sentence Summary: An exosite-binding inhibitor of ADAM10 is efficacious in the CIA model.

Supplemental Figures:

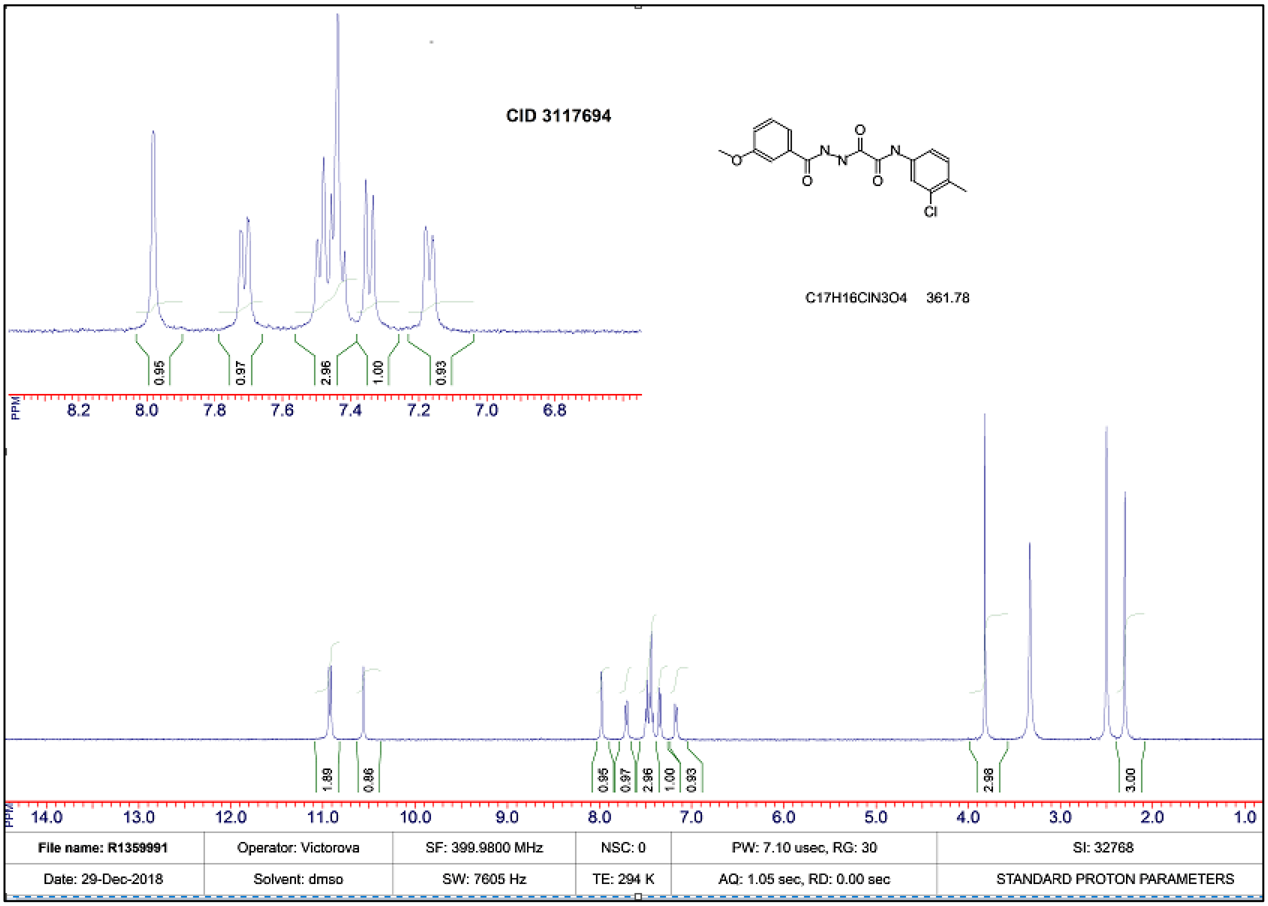

Supplemental Figure S1. Structural characterization of batch of CID 3117694 synthesized for this study by H1 NMR confirms the identity of the compound.

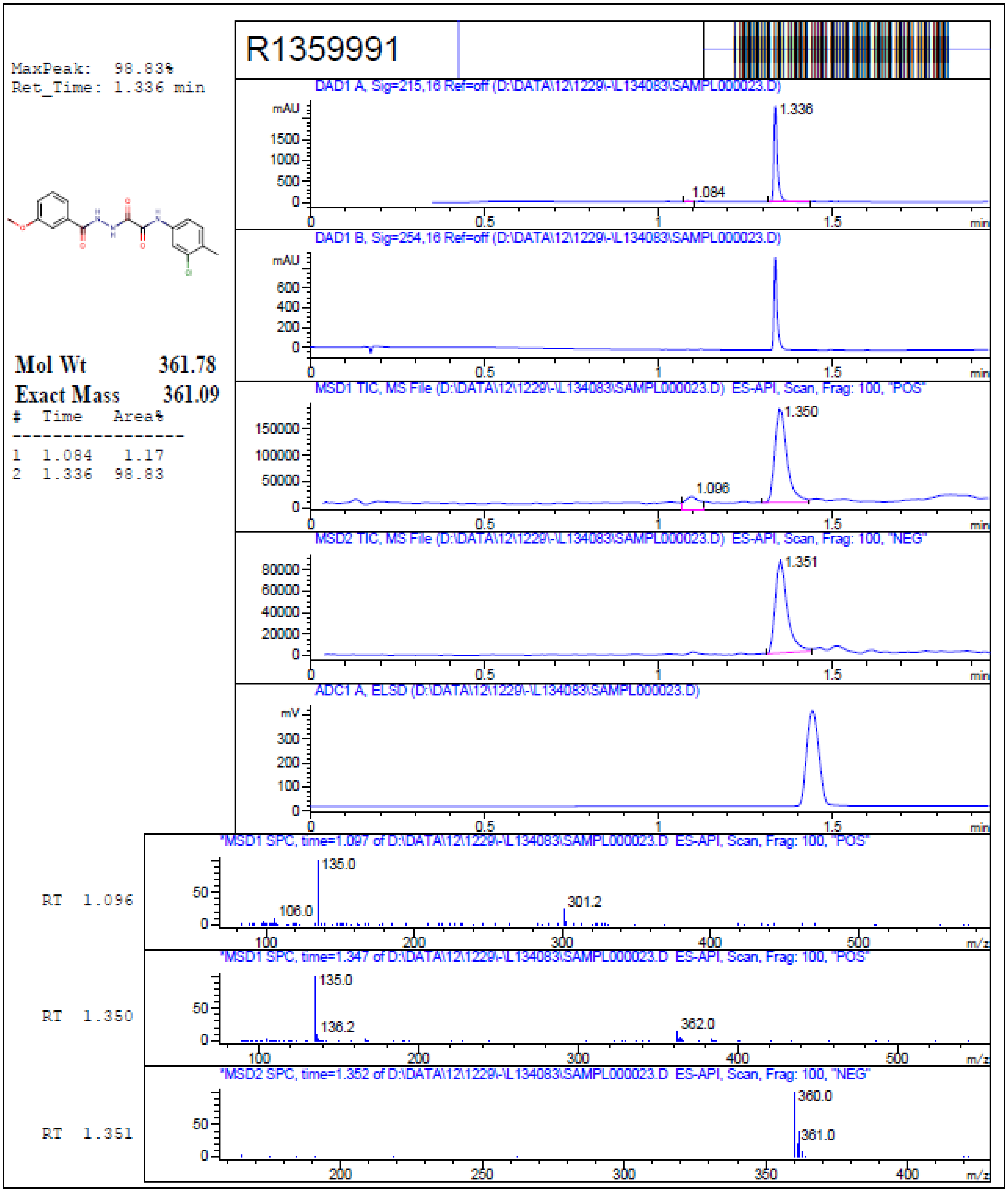

Supplemental Figure S2. **LC/MS/MS characterization of batch of CID 3117694 synthesized for this study confirms its purity and correct molecular weight.**
